# Combination of *Panax ginseng* and *Diospyros kaki* Leaf Inhibits White Adipocyte Differentiation and Browning Process through AMP-Activated Protein Kinase (AMPK) Activation In Vitro and In Vivo

**DOI:** 10.3390/nu15122776

**Published:** 2023-06-16

**Authors:** Hwa-Young Lee, Geum-Hwa Lee, Hwa-Jin Kim, Young Jae Lim, Bo Mi Ko, Do-Sung Kim, Tae Won Kim, Hye Kyung Kim, Tae Young Kim, Dae Il Hwang, Ha Kyoung Choi, Seon Min Ju, Kyung Hyun Min, Han-Jung Chae

**Affiliations:** 1 Non-Clinical Evaluation Center, Biomedical Research Institute, Jeonbuk National University Hospital, Jeonju 54907, Jeollabuk-do, Republic of Korea; youngat84@gmail.com (H.-Y.L.); tailove2212@naver.com (H.-J.K.); goboming@naver.com (B.M.K.);; 2 Research Institute of Clinical Medicine of Jeonbuk National University-Biomedical Research Institute of Jeonbuk National University Hospital, Jeonju 54907, Jeollabuk-do, Republic of Korea; heloin@jbnu.ac.kr; 3 College of Pharmacy, Kyungsung University, 309 Suyeong-ro, Nam-gu, Busan 48434, Republic of Koreafiona30@ks.ac.kr (H.K.K.); 4 Institute of Jinan Red Ginseng, Jinan-gun 55442, Jeollabuk-do, Republic of Korea; 5 School of Pharmacy, Jeonbuk National University, Jeonju 54896, Jeollabuk-do, Republic of Korea

**Keywords:** adipogenesis, browning, AMPK, sirtuin 1, obesity

## Abstract

Activating brown adipose tissue (BAT) and stimulating white adipose tissue (WAT) browning is a prospective obesity treatment method. Dietary components derived from plants are the most effective approach to activate BAT and promote WAT browning in rodents. This study investigated the synergistic effects of *Panax ginseng* (PG) and *Diospyros kaki* leaf (DKL) extract on adipocyte differentiation and browning, as well as the molecular mechanism underlying their beneficial effects. The administration of PG and DKL to HFD-induced obese mice significantly decreased body weight and epididymal and abdominal adipose tissue mass. In in vitro, PG inhibited the adipogenesis of 3T3-L1 adipocytes by regulating the expression of key adipogenic regulators, such as peroxisome proliferator-activated receptor (PPAR)γ and CCAAT/enhancer-binding protein (C/EBP)-α. In contrast, DKL negligibly influenced the adipogenesis of 3T3-L1 adipocytes but greatly increased the protein expression of UCP-1, PGC-1α, and PPARα in BAT and/or WAT. Moreover, PG and DKL inhibited adipogenesis synergistically and activated white adipocyte browning via AMP-activated protein kinase (AMPK) and sirtuin 1 (SIRT1) pathways. These results suggest that a combination of PG and DKL regulates adipogenesis in white adipocytes and browning in brown adipocytes by activating AMPK/SIRT1 axis. The potential use of PG and DKL may represent an important strategy in obesity management that will be safer and more effective.

## 1. Introduction

Obesity is a global epidemic that increases the risk of morbidity, reduces life expectancy, and is closely associated with increased risk of developing cardiometabolic disorders [1,2]. The evolution of two specialized adipose tissues has allowed mammals to acclimate to diverse metabolic demands. White adipose tissue (WAT) can store energy in the form of triglycerides, while brown adipose tissue (BAT) discharges energy in heat to regulate body temperature [3,4]. Typically, BAT is represented by a high density of mitochondria that express BAT-specific protein called uncoupling protein 1 (UCP-1) [5]. WAT is an endocrine tissue that secretes adipocytokines, essential for regulating various physiological functions. The released adipocytokines may contribute to the onset of metabolic syndrome under pathophysiological conditions [6,7,8]. Accumulating evidence suggests functional foods significantly regulate specific adipogenesis processes like white adipocyte differentiation or browning process. When functional foods recognized for their role in regulating the adipogenesis process are combined, resulting functional food complexes act synergistically to reduce health issues associated with the adipogenesis process. 

*Panax ginseng* is globally used to treat immune dysfunction, asthma, colds, fevers, flatulence, colic, diarrhea, and diabetes [9,10,11]. Previous pharmacological studies suggest that *Panax ginseng* has antioxidant and anti-inflammatory properties [12]. Specifically, *Panax ginseng* decreases lipid accumulation and hyperlipidemia in adipose tissue [13]. On the other hand, *Diospyros kaki* leaf is frequently used in traditional Chinese medicine to treat diabetes, oxidative stress, and inflammation [14,15,16]. Multiple reports on animal models indicate that the extracts of *Diospyros kaki* leaf suppress lipid accumulation in adipocytes and reduce pancreatic amylase, cholesterol, and triglyceride levels [17,18], suggesting a potential functional food against obesity and its associated metabolic disorder. These findings suggest that combining *Panax ginseng* and *Diospyros kaki* leaf may have a protective effect against lipid metabolism and adipogenesis, leading to greater therapeutic benefits than either extract alone. Hence, the present investigation aimed to determine the synergistic effects of *Panax ginseng* (PG) and *Diospyros kaki* leaf (DKL) extract on adipocyte differentiation and browning, as well as the molecular mechanism underlying their beneficial effects. To accomplish the objectives, individual in vitro tests were conducted with PG and DKL extracts to ascertain their synergistic effects on 3T3-L1 cells and the likely mechanism by which they exert their effects. Following in vitro tests, in vivo studies were conducted to determine the regulatory effects of PG and DKL on adipocyte differentiation and browning.

## 2. Materials and Methods

### 2.1. Preparation of Panax ginseng and Diospyros kaki Leaf Extract

Red ginseng and *Diospyros kaki* leaf were provided by institute of Jinan red ginseng (Jinan, Republic of Korea). Dried red ginseng (5 kg) and *Diospyros kaki* leaf (5 kg) were pulverized with a blender and extracted with 50 L of distilled water for 3 h, at 80 °C under reflux two times. Filtered red ginseng extract and *Diospyros kaki* leaf extract were concentrated using a vacuum rotary evaporator and lyophilized with a freezing dryer. Yield of red ginseng extract and *Diospyros kaki* leaf were 19.6% and 20.7 %, powdered red ginseng extract and *Diospyros kaki* leaf extract were stored at 4 °C until use. In this study, PG and DKL extracts were mixed in a 1:3 ratio to prepare the PG/DKL mixture, which was then prepared in three distinct concentrations.

### 2.2. HPLC Condition for Analysis

Ginsenoside Rg1, ginsenoside Rb1, ginsenoside Rg3 and tannic acid standards and samples were dissolved in 100% methanol and then used in HPLC analysis system (Hitachi High-Technologies Corporation, Tokyo, Japan). The HPLC analysis system was performed on a Zorbax Eclipse XDB-C18 (250 mm × 4.6 mm, 5 µm) analytical column equipped with acetonitrile in solvent (A) and distilled water in solvent (B). HPLC conditions for analysis of standards were shown in Supplementary Tables S1 and S2. 

### 2.3. Animal Experiment

Six-week-old male C57BL/6J mice were purchased from Orient Science Co. Seongnam, Republic of Korea. All the mice were housed at a designated animal laboratory. The environmental temperature was set at 22 ± 2 °C, and a 12 h L/D cycle was maintained. The mice were acclimated for a week on a regular chow diet. After a week of acclimatization, the animals were assigned to one of six groups that were weight matched. Each group consisted of 8 animals. Group 1, the normal diet (NCD) group, mice were fed a standard chow diet containing 11.5% fat, 20.8% protein, and 67.7% carbohydrates. Group 2, NCD mice supplemented with PG/DKL 200 mg/kg. Group 3, HFD group, mice fed with an HFD containing 60% fat, 20% protein, and 20% carbohydrates (Rodent Diet D12492, Research Diets, New Brunswick, NJ, USA). Group 4, HFD mice supplemented with PG/DKL 50 mg/kg. Group 5, HFD mice supplemented with PG/DKL 100 mg/kg. Group 6, HFD mice supplemented with PG/DKL 200 mg/kg. Mice were fed with NCD or HFD with vehicle or 50, 100, 200 mg/kg PG/DKL once daily for 9 weeks by oral gavage, and the body weight of the experimental animals was measured once a week for 9 weeks. Food intake was measured daily by cage and divided by the number of mice within the cage. At the end, all mice were euthanized following an intraperitoneal injection of 100 mg/kg ketamine (#VINB-KET0-7021, Henry Schein Animal Health, Dublin, OH, USA) with 10 mg/kg xylazine hydrochloride supplement (#X1251-1G, Sigma-Aldrich, St. Louis, MO, USA). Specifically, collected whole blood was stored at 2 °C for 30 min and then centrifuged to collect serum. All other samples were stored at −80 °C. Housing and handling of animals were done as suggested in the Jeonbuk National University Hospital animal care and used committee guidelines. The approval number assigned to this study is JBUH-IACUC-2021-4-1.

### 2.4. Serum Assay of Biochemical Parameter

At the end of the 11th week, the mice were fasted for 15 h prior to euthanasia. Blood samples were collected from a truncal vein and centrifuged at 1100× *g* for 15 min at 4 °C to separate serum and stored at −70 °C. The serum levels of triglyceride (TG), total cholesterol (T-Chol), low-density lipoprotein cholesterol (LDL-Chol), alanine aminotransferase (ALT), and aspartate aminotransferase (AST) and level in the liver tissue were analyzed using an automatic biochemical analyzer (Hitachi-7020, Hitachi Medical, Tokyo, Japan). The serum concentrations of leptin and adiponectin levels were assayed using mouse enzyme-linked immunosorbent assay (ELISA) kits (R & D Systems, Minneapolis, MN, USA).

### 2.5. Tissue Weight and Histological Analysis

Mice were dissected to collect abdominal subcutaneous fat (abdominal subWAT), epididymal WAT (eWAT), retroperitoneal WAT (rWAT), intestinal WAT, and the liver. Immediately after the collection, tissues were weighed and processed for further analysis. For adipocyte staining, the eWAT and liver were fixed with 10% neutral formalin and embedded in paraffin. All the tissue samples were sectioned at 6 μm and stained with hematoxylin and eosin (H & E). Images were captured using an inverted confocal microscope (Leica DMIRE2; Leica Microsystems, Wetzlar, Germany) with a 63× oil immersion objective lens. All the images were captured with the same laser intensities.

### 2.6. Adipocyte Size Measurement

Fixed adipose tissues were cut into 5 µm thick sections and stained with H & E. Microphotographs were captured at 200 magnification using Olympus BX51 microscope (M/S-Japan) fitted with DP 70 digital camera. The captured images were processed with the Image-Pro Plus version 6.0 application (Media Cybernetics, Rockville, MD, USA). Approximately 20% of the samples for morphometric analysis had fewer than 100 nonoverlapping adipocytes. Moreover, a comparison was made between the adipocyte measurements (mean area and diameter) obtained by analyzing 100 versus 50 adipocytes from the same individual to determine the differences. Observations revealed no statistically significant difference. Thus, 50 nonoverlapping adipocytes were used for measurements to ensure uniformity. The area and diameter of these adipocytes were calculated. All the data on area and diameter were compiled and exported to Microsoft Excel.

### 2.7. Oil Red O Staining

Oil red O staining was performed as reported earlier [19]. The 3T3-L1 and liver sections were washed, fixed with formaldehyde, and stained with 0.6% (*w*/*v*) Oil Red O solution at ambient temperature. Next, the tissue sections or cells were treated with 60% isopropanol to remove the unbound dye, and images were acquired using a Nikon microscope (Tokyo, Japan).

### 2.8. Western Blotting Analysis

Immunoblotting was performed as recommended in previous reports [20]. Briefly, protein samples were separated on a polyacrylamide gel and then transferred to PVDF membranes. Then, membranes were blocked with skimmed milk or 5% BSA and incubated with relevant primary antibodies. Next, the membrane was washed and incubated with species-specific secondary antibodies. All the signals were detected with the help of ECL solution. The antibodies used in the study were as follows: AMP-activated kinase (AMPK, #2532), phosphorylation of AMP-activated kinase (*p*-AMPK, P-2535), IRE1α (#3294), CHOP (#2895), ATGL (#2138), *p*-ACC (#11818), and ACC (#3676) from Cell Signaling Technologies, Inc., Danvers, MA, USA. Sterol regulatory element-binding protein (SREBP-1c, sc-36553), peroxisome proliferator-activated receptor α (PPAR-α), peroxisome proliferator-activated receptor γ (PPARγ), CCAAT/enhancer-binding protein α (C/EBP1α, sc-166258), fatty acid synthase (FAS, sc-74540), sirtuin 1 (SIRT1, sc-74465), GRP78 (#sc-13539), and β-actin (sc-47778) from Santa Cruz Biotechnology, USA. *p*-IRE1α (#ab124945) was purchased from Abcam, Cambridge, MA, USA. 

### 2.9. Quantitative Real-Time Polymerase Chain Reaction (qPCR)

The collected abdominal eWAT was homogenized, and proteins in the samples were denatured with TRI reagent (Sigma-Aldrich, St. Louis, MO, USA). Then, reverse transcription was done with oligo dT primers. The primer sequences used in this investigation are listed in Table 1. The obtained samples were digested with DNase I (Life Technologies, Grand Island, NY, USA) to remove the chromosomal DNA. Next, qRT-PCR was carried out with SYBR premix (TaKaRa Bio Inc., Shiga, Japan), and PCR was done with ABI PRISM 7500 Real-Time PCR system (Applied Biosystems, Foster City, CA, USA). For quantification, the comparative cycle threshold (Ct) method was adopted.

### 2.10. Dual-Energy X-ray Absorptiometry (DXA) Scan

DEXA scan was performed as described previously [21]. The fat and fat mass percentages were calculated with the help of a cone-beam flat panel detector DXA (iNSiGHT VET DXA, Osteosys, Daegu, Republic of Korea) following the manufacturer’s instructions. Fat mass was calculated by subtracting the lean body mass from the total body weight. Fat and lean tissue are indicated in red and green in full body composition images, respectively. The full body scan was used to determine the region of interest (ROI) to assess abdominal fat. The percentage of abdominal fat was estimated using the following equation, abdominal fat (DEXA)/total fat (DEXA) × 100.

### 2.11. Statistical Analysis

Data were analyzed using GraphPad Prism 8 software (GraphPad Prism 8, San Diego, CA, USA), and results were expressed as mean ± SEM. Data normality was assessed with the Shapiro–Wilk test. For normal data, equal-variance test was carried out by F test (for comparing two groups) or Bartlett’s test (for comparing more than 3 groups). When comparing 2 groups, either 2-tailed unpaired *t*-test (for parametric data) or the Mann–Whitney test (for nonparametric data) was used. When comparing more than 3 groups, one-way analysis of variance (ANOVA) (for parametric data) or the Kruskal–Wallis test (for nonparametric data) was carried out, followed by post-hoc multiple comparisons tests either by Dunnett, Tukey-Kramer, or Dunn’s test. *p*-values from these multiple comparison tests were corrected and reported as adjusted *p*-values. Statistical significance was set at *p* < 0.05. Each individual experiment was conducted concurrently with both HFD animals and their NCD-fed controls. No animals were excluded from analysis. Sample sizes (*n* = 8 mice) were determined a priori by estimating the effect size and data variability to yield a statistical power of 80% at α = 0.05. Sample sizes for each analysis are detailed in figure legends.

## 3. Results

### 3.1. Quantitative Analysis of Ginsenoside Rg1, Rb1, Rg3 and Tannic Acid in Panax ginseng (PG) and Diospyros kaki Leaf (DKL)

The retention times and chromatographs of standards were compared with those of PG, DKL, and their mixture (PG/DKL) to test analytical interference. As shown in Supplementary Figures S1 and S2, all of the standard peaks are separated well without interference by other peaks in PG, DKL, and their mixture (PG/DKL). The calibration curves of standards were calculated as regression equations (y = ax + b) and the correlation coefficients (R^2^) of standards were over 0.99 (Supplementary Table S3). Quantitative analysis of ginsenoside Rg1, Rb1, Rg3 and tannic acid in PG, DKL and their mixture (PG/DKL) were calculated using the linear calibration curves. In this study, we observed 0.87, 1.26, and 0.09% of Rg1, Rb1, and Rg3, respectively, in PG while tannic acid was 5.49% in DKL. In the PG/DKL mixture, Rg1, Rb1, Rg3, and tannic acid were 0.25, 0.23, 0.03, and 5.06%, respectively. Among the three kinds of ginsenosides components, contents of ginsenoside Rb1 was the highest, followed by ginsenoside Rg1 in PG and PG/DKL. Comparing contents of ginsenosides in PG and PG/DKL, the contents of ginsenosides in PG was measured about three times more than those in PG/DKL. On the contrary, contents of tannic acid in DKL and PG/DKL were high compared with contents of ginsenosides in PG and PG/DKL and the content of tannic acid in DKL was higher than that of tannic acid in PG/DKL (Supplementary Table S4).

### 3.2. Panax ginseng (PG) Extract and PG Active Compounds Regulate Adipogenesis in 3T3-L1 Cells

**Figure 1:**
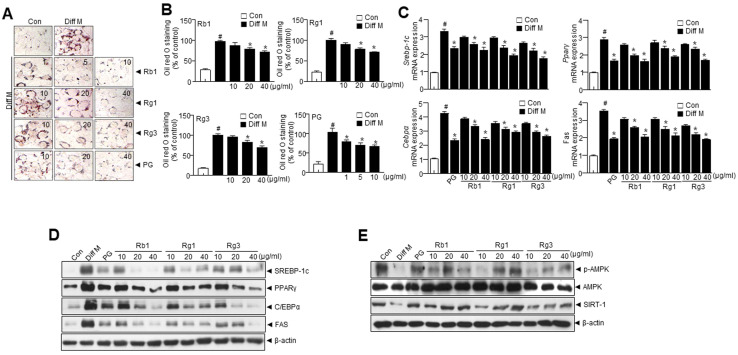
*Panax ginseng* (PG) and its active components regulate adipogenesis in 3T3-L1 cells. 3T3-L1 preadipocytes were induced to differentiation two days after 3T3-L1 cells reached full confluence, they were treated with a medium containing a differentiation cocktail containing MDI [10% FBS, 3-isobutyl-1-methylxanthine (0.5 mM), dexamethasone (1 μM), and insulin (10 μg/mL)] for two days, and then, for an additional three days, the cells were maintained in DMEM containing 10% FBS and insulin (10 μg/mL). (**A**) Representative images showing Oil Red O-stained cells treated with PG and its active compounds. (**B**) Quantification of lipid droplets. (**C**) mRNA expression levels of *Srebp-1c*, *Pparγ, Cebpα*, and *Fas*. (**D**) Immunoblotting of SREBP-1c, PPARγ, C/EBPα, FAS, and β-actin. (**E**) Immunoblotting of *p*-AMPK, AMPK, SIRT1, and β-actin. Data are presented as mean ± SEM. (# *p* < 0.05 vs. Con, * *p* < 0.05 vs. Diff M). Con; control, Diff M; differentiation media.

To evaluate the anti-adipogenic effects of *Panax ginseng* (PG) and its active compounds, Rb1, Rg1, and Rg3, 3T3-L1 preadipocytes were treated with PG extracts, Rb1, Rg1, and Rg3 for seven days. The differentiation of preadipocytes into adipocytes is associated with lipid accumulation, indicated by the increased number of Oil red O-stained cells. Thus, 3T3-L1 preadipocytes were treated with PG and stained with Oil Red O. Treatment with extracts and active compounds significantly reduced the accumulation of intracellular lipids (Figure 1A,B).

To investigate the role of adipocyte-specific transcription factors on 3T3-L1 preadipocyte differentiation, cells treated with PG and its active compounds were examined for SREBP-1c, PPARγ, C/EBPα, and FAS expression at transcriptional and translational levels. PG and its active compounds significantly reduced the expression of adipogenic transcription factors in 3T3-L1 adipocytes (Figure 1C,D). Further, phosphorylation of AMPK and SIRT1 were relatively less expressed in the Diff M, while PG and its active compound treatment significantly enhanced *p*-AMPK and SIRT1 expression (Figure 1E).

### 3.3. Diospyros kaki Leaf (DKL) Extract and DKL Active Compounds Induce Browning in 3T3-L1 Adipocytes

**Figure 2:**
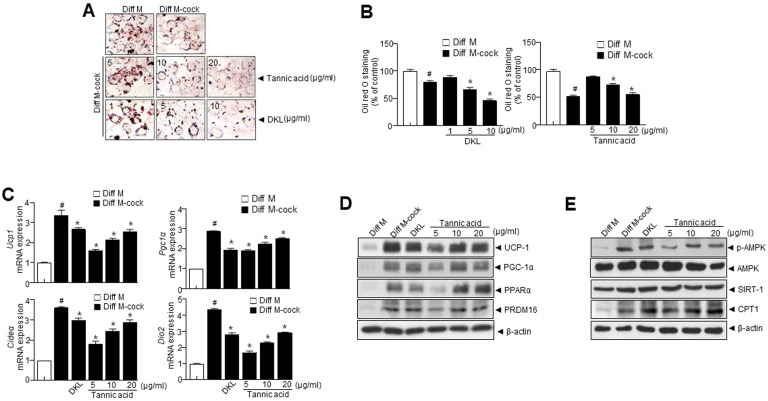
*Diospyros kaki* leaf (DKL) and its active compounds induce browning in 3T3-L1 adipocytes. Browning was induced 3T3-L1 preadipocytes after 3T3-L1 cells reached full confluence, they were treated with medium containing a differentiation cocktail containing 50 nM triiodothyronine, and maturation medium was supplemented with 50 nM triiodothyronine and 1 μM rosiglitazone. (**A**) Representative images showing Oil Red O-stained cells treated with DKL and its active compounds. (**B**) Quantification of lipid droplets. (**C**) Immunoblotting of UCP-1, PGC-1α, PPARα, PREM16, and β-actin expressions in 3T3-L1. (**D**) mRNA expression levels of *Ucp1*, *Pgc1α*, *Cideα*, and *Dio2*. (**E**) Immunoblotting of *p*-AMPK, AMPK, SIRT1, CPT1, and β-actin. Data are presented as mean ± SEM. (# *p* < 0.05 vs. Con, * *p* < 0.05 vs. Diff M). Diff M; differentiation media, Diff M-cock; differentiation media cocktail.

Next, we sought to investigate the effects of DKL and tannic acid on the brown fat-like phenotype in 3T3-L1 cells. Cells were treated with a browning cocktail (50 nM triiodothyronine and 1 μM rosiglitazone) and treated with DKL and different doses of tannic acid. When 3T3-L1preadipocytes were subjected to the differentiation medium, they differentiated into adipocytes and induced the accumulation of lipid droplets. Hence, cells treated with a browning cocktail and treated with DKL and different doses of tannic acid significantly suppressed lipid accumulation (Figure 2A,B). DKL and tannic acid markedly upregulated the brown fat markers such as *Ucp1*, *Pgc1α*, *Cideα*, and *Dio2* (Figure 2C). Further, DKL and tannic acid treatment resulted in the upregulation of genes specific to brown fat (Figure 2D). Additionally, DKL and tannic acid significantly elevated *p*-AMPK, SIRT1, and CPT1 protein levels (Figure 2E), suggesting enhanced lipolysis, fatty acid oxidation, and mitochondrial biogenesis. 

### 3.4. Synergistic Effect of Panax ginseng (PG) and Diospyros kaki Leaf (DKL) Extracts Regulate Lipid Accumulation and Browning of 3T3-L1 White Adipocytes

**Figure 3:**
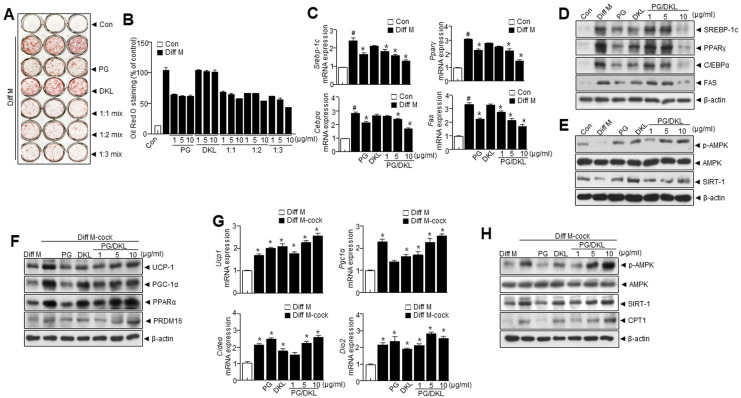
The combination of *Panax ginseng* (PG) and *Diospyros kaki* leaf (DKL) regulates adipogenesis of white adipose tissue in 3T3-L1 cells. (**A**) Representative images showing Oil Red O-stained cells treated with different doses of PG/DKL. (**B**) Quantification of lipid droplets. (**C**) mRNA expression levels of *Srebp-1c*, *Pparγ, Cebpα*, and *Fas*. (**D**) Immunoblotting of SREBP-1c, PPARγ, C/EBPα, FAS, and β-actin expressions in 3T3-L1. (**E**) Immunoblotting of *p*-AMPK, AMPK, SIRT1, and β-actin expressions in 3T3-L1. (**F**) Immunoblotting of UCP-1, PGC-1α, PPARα, PREM16, and β-actin expressions in 3T3-L1. (**G**) mRNA expression levels of levels of *Ucp1*, *Pgc1α*, *Cideα,* and *Dio2*. (**H**) Immunoblotting of *p*-AMPK, AMPK, SIRT1, and β-actin expressions in 3T3-L1. Data are presented as mean ± SEM. (# *p* < 0.05 vs. Con, * *p* < 0.05 vs. Diff M). Diff M; differentiation media, Diff M-cock; differentiation media cocktail.

Generally, the combination of functional foods results in larger health benefits than using a single functional food. Thus, PG and DKL extracts were combined and their anti-adipogenic effects in 3T3-L1 cells were investigated. PG/DKL treatment reduced lipid accumulation (Figure 3A,B). Next, the effects of PG/DKL on lipid metabolism were investigated. Cells treated with PG/DKL effectively regulated the expression of adipogenic transcription factors like *Srebp1*, *Pparγ*, *Cebpα*, and *Fas* (Figure 3C,D). Interestingly, PG/DKL treatment significantly elevated *p*-AMPK and SIRT1, indicating enhanced lipolysis, fatty acid oxidation, and mitochondrial biogenesis (Figure 3E). Further, cells were treated with a browning cocktail (50 nM triiodothyronine and 1 μM rosiglitazone) and treated with PG/DKL. Here, brown fat markers such as PGC-1α, UCP-1, PPARα, and PRDM16 were markedly upregulated with PG/DKL treatment (Figure 3F–H). Together, these results indicate that a combination of PG and DKL has significant anti-adipogenic effects on 3T3-L1 preadipocytes.

### 3.5. Panax ginseng (PG) and Diospyros kaki Leaf (DKL) Extracts Synergistically Regulate Body Weight Gain and Their Effect on Metabolic Profile in HFD-Induced Obese Mice

**Figure 4:**
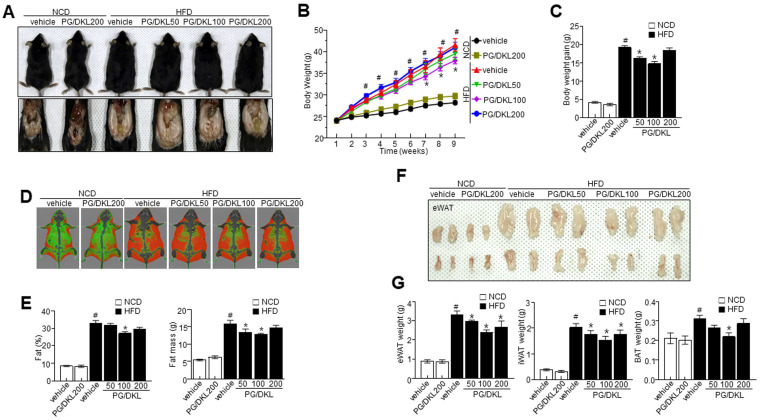
Synergistic effect of *Panax ginseng* (PG) and *Diospyros kaki* leaf (DKL) on body weight gain and its effect on metabolic profile in HFD-induced obese mice. Mice were fed with NCD or HFD with vehicle or 50, 100, 200 mg/kg PG/DKL once daily for nine weeks by oral gavage. (**A**) Representative images showing the physical appearance and fat accumulation, (**B**,**C**) analysis of body weight and body weight gain, (**D**) representative Dual-energy X-ray Absorptiometry (DXA) scan images, (**E**) visceral fat percentage and respective orbital fat mass, (**F**) representative images showing eWAT, (**G**) eWAT weight, iWAT weight, and BAT weight. Data are presented as mean ± SEM (*n* = 8, # *p* < 0.05 vs. NCD + vehicle, * *p* < 0.05 vs. HFD + vehicle).

In order to know the effect of PG/DKL against obesity, we applied doses of 50, 100, and 200 mg/kg at the established ratio, 1:3 of PG and DKL to normal chow diet (NCD) and high fat diet (HFD)-feeding mice. The supplementation of PG/DKL to HFD-induced obese mice showed reduced body weight compared to the HFD group (Figure 4A–C). Mice supplemented with 50 and 100 mg/kg of PG/DKL showed a greater body weight reduction than those supplemented with 200 mg/kg of PG/DKL. Dual-energy X-ray Absorptiometry (DXA) scan suggests higher visceral adipose tissue in the HFD group than in NCD and PG/DKL groups. The visceral adipose tissue of obese mice treated with PG/DKL was significantly reduced compared to their body weight (Figure 4D). Consistently, volume measurements of DXA scan confirm the reduction of orbital fat volume (%) and mass (g) with PG/DKL supplementation (Figure 4E). Similarly, epididymal white adipose tissue (eWAT), inguinal white adipose tissue (iWAT), and brown adipose tissue (BAT) was reduced (Figure 4F,G). Moreover, food intake was similar between NCD, HFD, and HFD supplemented with PG/DKL (Supplementary Figure S3). Further, hepatic enzymes were analyzed to determine the effect of PG/DKL on hepatic function. The supplementation of PG/DKL regulated the hepatic enzyme activities in HFD mice (Table 2). Further, supplementation of PG/DKL effectively regulated the LDL-cholesterol, triglycerides, adiponectin, and leptin levels, indicating the beneficial effect on lipid homeostasis (Table 2). However, in this study, there was no difference about obesity at the highest dose, 200 mg/kg of PG/DKL-administered NCD feeding mice compared with NCD-control mice (Figure 4). Further the highest dose of PG/DKL had no effect about hepatic enzyme, AST and ALT and lipid profile at NCD mice, indicating at least the dose 200 mg/kg did not show either toxicity or dysmetabolic effect (Table 2).

### 3.6. Panax ginseng (PG) and Diospyros kaki Leaf (DKL) Extracts Synergistically Regulate the Levels of Adipogenesis-Related Proteins in Adipose Tissues of HFD-Induced Obese Mice

**Figure 5:**
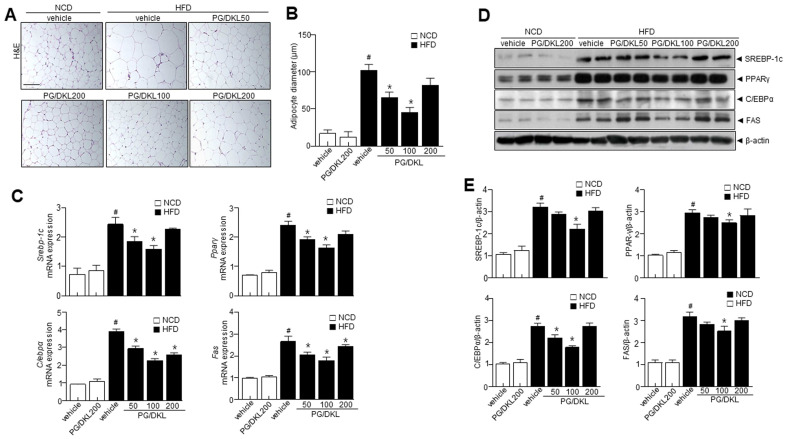
Synergistic effects of *Panax ginseng* (PG) and *Diospyros kaki* leaf (DKL) in determining adipose tissue expansion and adipogenic factors in eWAT. Mice were fed with NCD or HFD with vehicle or 50, 100, 200 mg/kg PG/DKL once daily for nine weeks by oral gavage. (**A**) H & E staining in eWAT and the average diameter of adipocytes in eWAT. Scale bar = 50 µm. (**B**) Measurements of adipocytes size. (**C**) mRNA expression levels of *Srebp-1c*, *Pparγ, Cebpα*, and *Fas*. (**D**,**E**) Immunoblotting of SREBP-1c, PPARγ, C/EBPα, FAS, and β-actin expressions and their quantification. Data are presented as mean ± SEM (*n* = 8, #*p* < 0.05 vs. NCD + vehicle, * *p* < 0.05 vs. HFD + vehicle).

**Figure 6:**
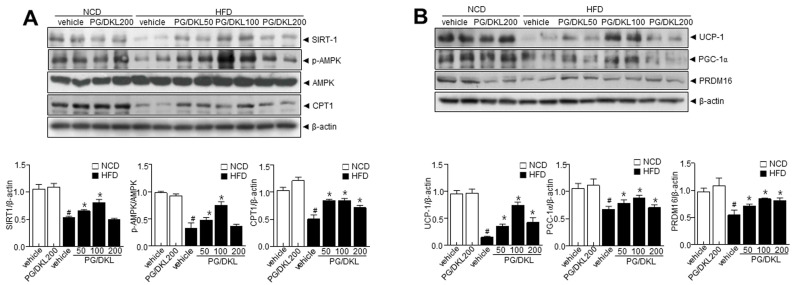
Synergistic effects of *Panax ginseng* (PG) and *Diospyros kaki* leaf (DKL) promote thermogenic program in adipose tissue. Mice were fed with NCD or HFD with vehicle or 50, 100, 200 mg/kg PG/DKL once daily for nine weeks by oral gavage. (**A**) Immunoblotting using antibodies against SIRT1, *p*-AMPK, AMPK, CPT1, and β-actin in eWAT and respective quantitative analysis. (**B**) Immunoblotting using antibodies against UCP-1, PGC-1α, PRDM16, and β-actin in the eWAT and respective quantitative analysis. Data are presented as mean ± SEM (*n* = 8, # *p* < 0.05 vs. NCD + vehicle, * *p* < 0.05 vs. HFD + vehicle). Epididymal white adipose tissue; eWAT.

We assessed the adipogenesis factors to verify the correlation between hepatic metabolism and adipogenesis factors with PG/DKL supplementation. H & E staining revealed that the PG/DKL-treated condition is highly efficient in reducing the size and diameter of the adipocytes compared to the HFD group (Figure 5A,B). Interestingly, genes associated with fatty acid biosynthesis were higher in the HFD group than in NCD. However, mice supplemented with PG/DKL showed reduced fatty acid biosynthesis genes (Figure 5C). Further, PG/DKL supplementation reduced the expressions of adipogenic transcription factors in eWAT (Figure 5D,E). The reduction in adipogenic transcription factors suggests that PG/DKL regulates eWAT weight by altering adipogenesis-associated transcription factors. Next, we evaluated the expression of upstream AMPK and SIRT1. AMPK plays a critical role in regulating fatty acid metabolism and in the development of adipose tissue, its activation correlates with decreased lipid storage [22], and SIRT1 is a critical regulator of AMPK signaling in controlling lipid metabolism. HFD mice showed reduced AMPK phosphorylation without affecting total AMPK levels, resulting in a decrease in the *p*-AMPK/AMPK ratio, which was recovered by PG/DKL treatment. Similarly, modulation of SIRT1 was observed upon PG/DKL treatment (Figure 6A,B). In this study, there was no difference in adipose tissue-derived adipogenesis parameters, including SREBP-1c, PPAR-γ, C/EBPα, and FAS and metabolic reprogramming genes, including SIRT-1, *p*-AMPK, CPT1, UCP-1, PGC-1α, and PRDM16 at the highest dose, 200 mg/kg of PG/DKL-administered NCD feeding mice compared with NCD-control mice (Figure 5 and Figure 6).

### 3.7. Combination of Panax ginseng (PG) and Diospyros K kaki Leaf (DKL) Extracts Ameliorate Hepatic Lipid Accumulation in the Liver and Brown Fat-like Phenotype in Adipose Tissue of HFD-Induced Obese Mice

**Figure 7:**
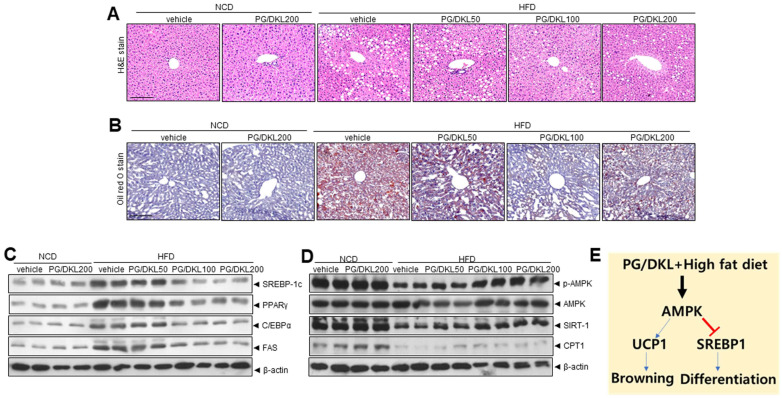
Synergistic effect of *Panax ginseng* (PG) and *Diospyros kaki* leaf (DKL) regulate hepatic lipid accumulation in HFD model. (**A**) Representative images of liver sections stained with H & E. Scale bars = 50 μm. (**B**) Representative images of liver sections stained with oil red O stain. (**C**) Immunoblotting of SREBP-1c, PPARγ, C/EBPα, FAS, and β-actin expressions. (**D**) Immunoblotting of *p*-AMPK, AMPK, SIRT-1, CPT1, and β-actin expressions. (**E**) Schematic diagram showing potential regulatory mechanisms in adipogenesis by of *Panax ginseng* (PG) and *Diospyros kaki* leaf (DKL) in adipose tissue.

eWAT expansion is known to cause ectopic fat accumulation in the liver. Thus, morphological investigations were done to assess the impact of PG/DKL on liver tissue. Histopathological analysis of the liver tissues indicated that PG/DKL supplementation substantially reduced the size and number of lipid droplets in liver tissues of HFD-induced obese mice (Figure 7A,B). Moreover, SREBP-1, C/EBPα, PPARγ, and FAS protein expressions were significantly higher in HFD mice than in the NCD group. However, supplementation with PG/DKL decreased the expression of these proteins that regulate fatty acid biosynthesis (Figure 7C). Additionally, *p*-AMPK and SIRT1 expressions were reduced in the HFD mice compared to in the NCD mice. However, PG/DKL reversed the effect by increasing expressions of *p*-AMPK and SIRT1 (Figure 7D), suggesting enhanced lipolysis, fatty acid oxidation, and mitochondrial biogenesis. In this study, there was no difference in hepatic lipogenesis parameters, including SREBP-1c, PPARγ, C/EBPα, and FAS and metabolic reprogramming genes, including *p*-AMPK, SIRT1 and CPT1 at the highest dose, 200 mg/kg of PG/DKL-administered NCD feeding mice compared with NCD-control mice (Figure 7).

## 4. Discussion

Activating brown adipose tissue (BAT) and stimulating white adipose tissue (WAT) browning is a prospective obesity treatment method. PG and DKL are known to prevent lipid metabolism and adipogenesis in white adipocytes. The study confirms the individual roles of PG and DKL in regulating adipogenesis and browning, respectively. Most importantly, the study evaluates the synergistic effect of PG/DKL against adipocyte differentiation and browning. The study also demonstrated the molecular mechanism by which PG and DKL extract regulates white adipocyte differentiation and the browning process in adipocytes (Figure 7E). PG extract significantly inhibited lipid accumulation during 3T3-L1 adipocyte differentiation with decreasing SREBP-1C, PPARγ, C/EBPα, and FAS expressions (Figure 1A–E). The efficacy of PG against obesity has been extensively studied and reported [23]. Previously, ginsenosides like Rb1, Rg1, and Rg3 were reported in PG, which inhibited adipogenesis by downregulating several adipogenic factors, including leptin and aP2, and transcription factors like C/EBPα, PPARγ, SREBP-1c, and Glut4 and by modulating the histone deacetylase sirtuin 1 [24,25,26]. Similarly, the present study was designed to evaluate the impact of PG. In vitro studies showed that ginsenosides, including Rg1 and Rg2, inhibited lipid accumulation throughout the whole process of adipogenesis differentiation [27,28,29]. 

DKL extract enhanced the expression level of UCP-1, PGC-1α, PPARα, and PRDM16, a marker of brown adipocytes in 3T3-L1 adipocytes (Figure 2A–E). Browning was enhanced after treatment with DKL extract, which may be the leading cause of the subsequent cascade effects, such as a decrease in cellular cytokine secretion. The ability of BAT to dissipate energy as heat drives the anti-inflammatory actions of BAT, where UCP-1 mediates heat dissipation [30] and regulates adipose tissue thermogenesis. The increase in cellular respiration has beneficial effects on other cellular pathways, such as AMPK-SIRT1 (Figure 1E and Figure 2E), which are essential for activating lipid metabolism, ultimately resulting in reducing obesity. 

PG extract regulates white adipocyte differentiation, while DKL extracts regulate adipocyte browning. The combination of PG/DKL demonstrated synergistic effects in regulating body weight, hepatic enzymes, lipid accumulation, adipogenic transcription factors, and browning. Importantly, AMPK plays a crucial function in adipocyte differentiation and browning, offering a key mechanism for understanding the synergistic effect of PG/DKL.

In this study, AMPK and its downstream signals, like UCP-1 and SREBP1, confirm the significance of AMPK in PG/DKL protective role against adipocyte differentiation and browning. Recently, our study mentioned high antioxidant effect controls SIRT1 decay, leading to stabilize the maintenance of SIRT1 expression [20,31]. The expression of SIRT1 was analyzed to determine whether it regulates genes linked to adipogenesis, lipogenesis, and fatty acid oxidation. Observations from this study confirm the effect of PG/DKL on SIRT1 expression, suggesting the involvement of the AMPK-SIRT1 axis in the protective effect of PG/DKL. Moreover, PG/DKL markedly lowered body weight gain and fat accumulation, indicating the involvement of PPARγ and C/EBPα and their downstream FAS (Figure 5C,D). Further, other transcriptional factors, such as CREB1 and SREBP-1c also correspond to the modulation of adipogenesis [32]. Reportedly, adipocyte differentiation requires SREBP1c, which can induce PPARγ and FAS expression [32]. Consistently, PG/DKL significantly attenuated SREBP-1c in the HFD group (Figure 5E). 

### Limitations of the Study

Additional confirmatory in vivo tests with AMPK knockout (KO) mice and in vitro cellular model application with AMPK knockdown technologies are necessary to confirm the suggested mechanism behind PG/DKL anti-obesity effects. However, an organized study based on the suggested pathway (adipocyte differentiation and browning through AMPK activation) may clarify the issue. Gene knockout investigations must be performed, especially with AMPK knockdown, to confirm therapeutic effect against obesity. Moreover, it is necessary to investigate whether the synergistic effects of PG/DKL would be the same in mice if the obesity model were first induced by a HFD, and then the animals were treated with PG/DKL, the other limitation to be overcome in the near future. 

## 5. Conclusions

Collectively, the findings of the study suggest the involvement of AMPK signaling; AMPK-SIRT1 and AMPK-UCP-1. However, further study is required to determine the signaling mechanism that may be implicated in addition to AMPK. In conclusion, PG/DKL regulates adipogenesis and browning by activating AMPK/SIRT1 axis. The potential use of PG and DKL may represent an important strategy in obesity management that will be safer and more effective.

## Figures and Tables

**Table 1:** Oligonucleotide and probe sequences.

Name	Forward (5′–3′)	Reverse (3′–5′)
*Srebp-1c*	CCATGGATGCACTTTCGAA	CCAGCATAGGGTGGGTCAA
*Fas*	CGGTACGCGACGGCTGCCTG	GCTGCTCCACGAACTCAAACACCG
*Pparγ*	GCAGCTACTGCATGTGATCAAGA	GTCAGCGGGTGGGACTTTC
Cebpα	GACTTGGTGCGTCTAAGATGAG	TAGGCATTGGAGCGGTGAG
*Ucp-1*	GGCCTCTACGACTCAGTCCA	TAAGCCGGCTGAGATCTTGT
*Pgc1* *α*	GAAAGGGCCAAACAGAGAGA	GTAAATCACACGGCGCTCTT
*Cidea*	GCCTGCAGGAACTTATCAGC	GCCTGCAGGAACTTATCAGC
*Dio2*	CTGCGCTGTGTCTGGAAC	GGAATTGGGAGCATCTTCAC
*β*-actin	CCTGGCACCCAGCACAAT	GCCGATCCACACACGGAGTACT

**Table 2:** *Panax ginseng* (PG) and *Diospyros kaki* leaf (DKL) extracts have a synergic effect on decreasing the biochemical markers of obesity.

Parameter	Group					
	NCD + Vehicle	NCD + PG/DKL200	HFD + Vehicle	HFD + PG/DKL50	HFD + PG/DKL100	HFD + PG/DKL200
ALT (U/L)	24.75 ± 4.47	26.25 ± 5.61	66.5 ± 7.31 ^#^	59.13 ± 3.37 *^#^	58.25 ± 8.14 *^#^	59.00 ± 4.61 *^#^
AST (U/L)	54.38 ± 6.38	58.50 ± 4.27	113.00 ± 14.87 ^#^	98.50 ± 6.12 *^#^	88.38 ± 6.14 *^#^	86.75 ± 6.16 *^#^
TG (mg/dL)	41.84 ± 13.47	45.04 ± 6.87	99.18 ± 15.32 *^#^	84.68 ± 13.67 *^#^	74.36 ± 14.17 *^#^	82.48 ± 11.14 *^#^
T-Chol (mg/dL)	77.63 ± 9.73	73.75 ± 11.40	142.63 ± 14.15 ^#^	135.75 ± 15.28 *^#^	122.38 ± 13.58 *^#^	125.13 ± 15.49 *^#^
LDL-Chol	16.40 ± 1.13	18.00 ± 3.64	131.75 ± 13.08 ^#^	102.25 ± 11.37 *^#^	89.75 ± 6.10 *^#^	106.50 ± 13.55 *^#^
Adiponectin (μg/mL)	10.38 ± 2.45	10.25 ± 2.90	5.58 ± 1.52 *^#^	6.14 ± 1.21 *^#^	8.04 ± 1.43 *^#^	7.25 ± 1.61
Leptin (ng/mL)	9.50 ± 3.61	7.25 ± 2.49	50.75 ± 6.74 ^#^	45.88 ± 4.91 *^#^	35.38 ± 5.10 *^#^	40.50 ± 7.86 *^#^
Liver TG(mg/g)	27.50 ± 6.30	23.50 ± 10.10	70.88 ± 12.57 ^#^	64.75 ± 8.94 *^#^	61.88 ± 12.54 *^#^	62.88 ± 6.57 *^#^

Note: The data are presented as mean ± SEM (*n* = 8). Data are presented as mean ± SEM (*n* = 8, ^#^ *p* < 0.05 vs. NCD + vehicle, * *p* < 0.05 vs. HFD + vehicle). ALT: Alanine aminotransferase, AST: Aspartate aminotransferase, TG: Triglyceride, T-Chol: Total cholesterol, LDL-Chol: Low Density Lipoprotein Cholesterol.

## Data Availability

The data presented in this study are available upon request from the corresponding author.

## Supplementary Materials

The following supporting information can be downloaded at: https://www.mdpi.com/article/10.3390/nu15122776/s1, Figure S1: Structure and chromatogram of ginsenoside Rg1, Rb1, and Rg3 in PG (A–C) and tannic acid in DKL respectively; Figure S2: Chromatogram of ginsenoside Rg1, Rb1, and Rg3 and tannic acid in mixture PG and DKL (1:3) respectively; Figure S3: The measurement of food intake in vehicle and 200 mg/kg mixture of PG and DKL (1:3)-administered NCD mice and in vehicle and 50, 100, and 200 mg/kg mixture of PG and DKL (1:3)-administered HFD mice during nine weeks; Table S1: HPLC condition for analysis of ginsenoside Rg1, Rb1, and Rg3 in PG; Table S2: HPLC condition for analysis of tannic acid in DKL; Table S3: Calibration data of ginsenoside Rg1, Rb1, and Rg3 in PG and tannic acid in DKL; Table S4: Contents of ginsenoside Rg1, Rb1, and Rg3 in PG and tannic acid in DKL.
