# Supplementary material for: Combination of Panax ginseng and Diospyros kaki Leaf Inhibits White Adipocyte Differentiation and Browning Process through AMP-Activated Protein Kinase (AMPK) Activation In Vitro and In Vivo
Source: Nutrients. 2023 Jun 16;15(12):2776. doi: 10.3390/nu15122776 (PMC10304786; doi:10.3390/nu15122776)
Supplement: Supplementary file 1 [file nutrients-15-02776-s001.zip › SUPPLE figure.pptx]

## Slide 1
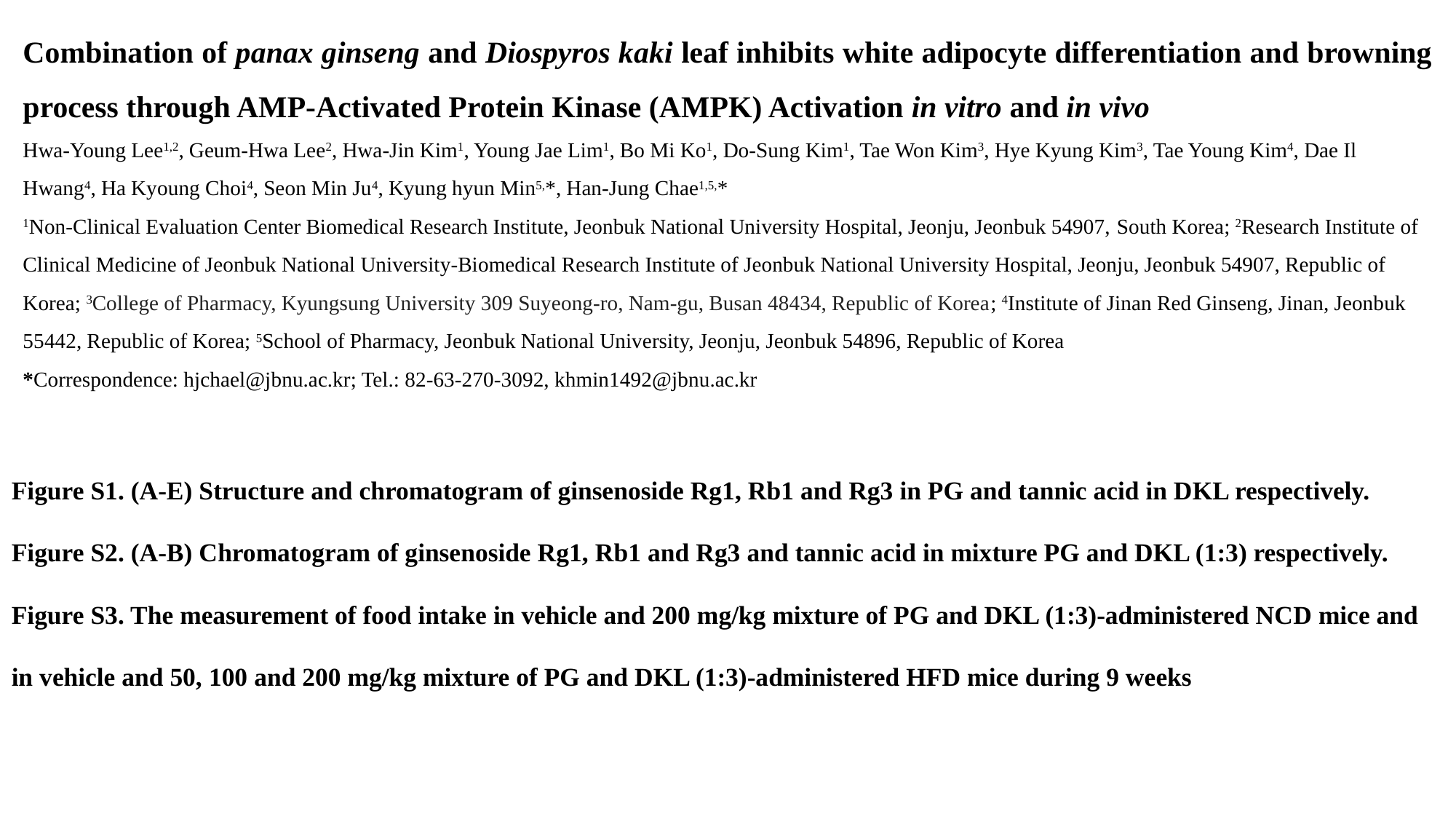

Combination of panax ginseng and Diospyros kaki leaf inhibits white adipocyte differentiation and browning process through AMP-Activated Protein Kinase (AMPK) Activation in vitro and in vivo
Hwa-Young Lee1,2, Geum-Hwa Lee2, Hwa-Jin Kim1, Young Jae Lim1, Bo Mi Ko1, Do-Sung Kim1, Tae Won Kim3, Hye Kyung Kim3, Tae Young Kim4, Dae Il Hwang4, Ha Kyoung Choi4, Seon Min Ju4, Kyung hyun Min5,*, Han-Jung Chae1,5,*
1Non-Clinical Evaluation Center Biomedical Research Institute, Jeonbuk National University Hospital, Jeonju, Jeonbuk 54907, South Korea; 2Research Institute of Clinical Medicine of Jeonbuk National University-Biomedical Research Institute of Jeonbuk National University Hospital, Jeonju, Jeonbuk 54907, Republic of Korea; 3College of Pharmacy, Kyungsung University 309 Suyeong-ro, Nam-gu, Busan 48434, Republic of Korea; 4Institute of Jinan Red Ginseng, Jinan, Jeonbuk 55442, Republic of Korea; 5School of Pharmacy, Jeonbuk National University, Jeonju, Jeonbuk 54896, Republic of Korea
*Correspondence: hjchael@jbnu.ac.kr; Tel.: 82-63-270-3092, khmin1492@jbnu.ac.kr
Figure S1. (A-E) Structure and chromatogram of ginsenoside Rg1, Rb1 and Rg3 in PG and tannic acid in DKL respectively.
Figure S2. (A-B) Chromatogram of ginsenoside Rg1, Rb1 and Rg3 and tannic acid in mixture PG and DKL (1:3) respectively.
Figure S3. The measurement of food intake in vehicle and 200 mg/kg mixture of PG and DKL (1:3)-administered NCD mice and in vehicle and 50, 100 and 200 mg/kg mixture of PG and DKL (1:3)-administered HFD mice during 9 weeks

## Slide 2
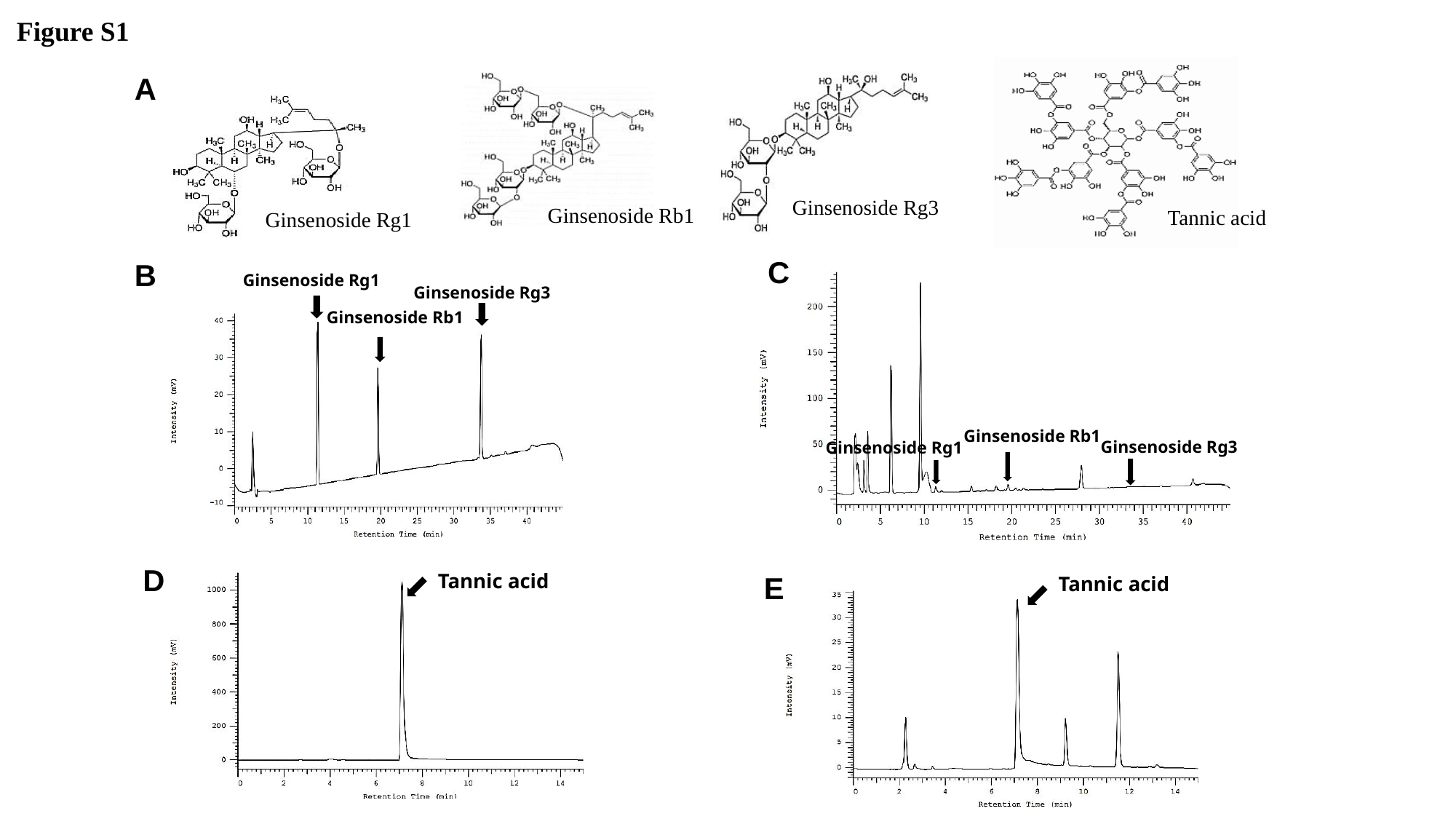

Figure S1
A
Ginsenoside Rg3
Ginsenoside Rb1
Tannic acid
Ginsenoside Rg1
C
B
Ginsenoside Rg1
Ginsenoside Rb1
Ginsenoside Rg3
Ginsenoside Rg1
Ginsenoside Rg3
Ginsenoside Rb1
D
Tannic acid
E
Tannic acid

## Slide 3
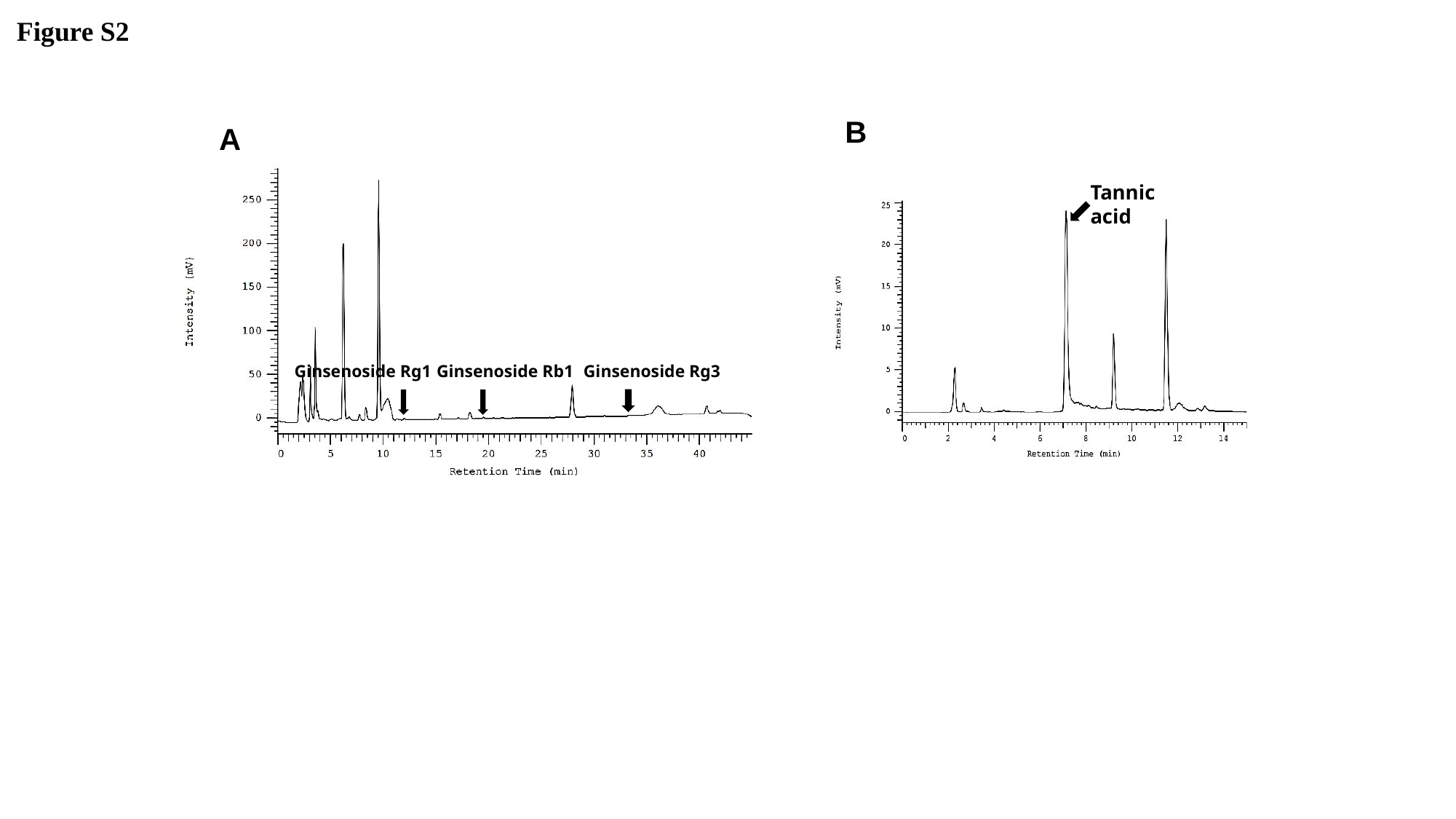

Figure S2
B
A
Tannic acid
Ginsenoside Rg1
Ginsenoside Rb1
Ginsenoside Rg3

## Slide 4
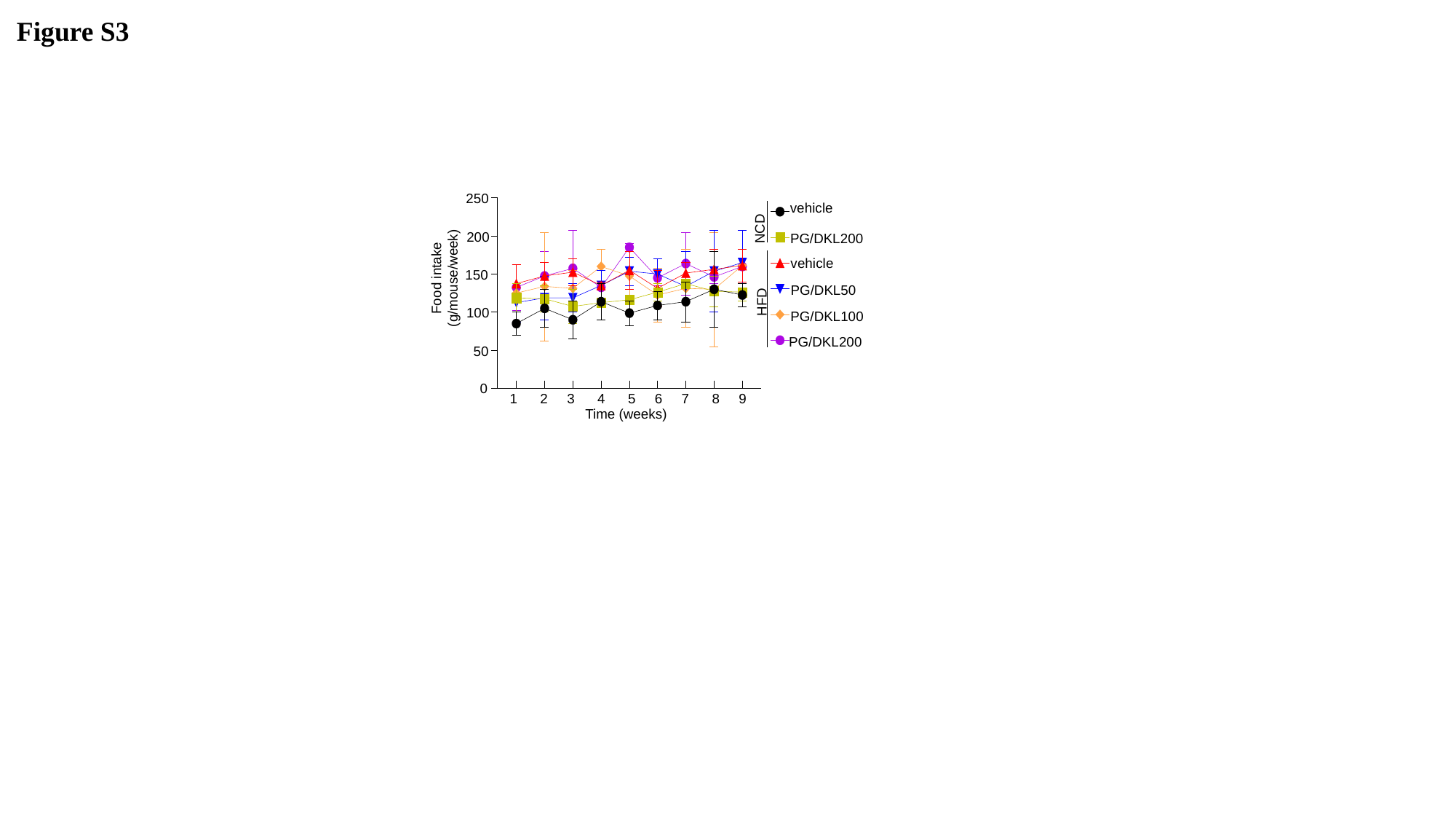

Figure S3
250
vehicle
NCD
200
PG/DKL200
 vehicle
Food intake
(g/mouse/week)
150
PG/DKL50
HFD
100
PG/DKL100
PG/DKL200
50
0
1 2 3 4 5 6 7 8 9
 Time (weeks)
