# Supplementary material for: Combination of Panax ginseng and Diospyros kaki Leaf Inhibits White Adipocyte Differentiation and Browning Process through AMP-Activated Protein Kinase (AMPK) Activation In Vitro and In Vivo
Source: Nutrients. 2023 Jun 16;15(12):2776. doi: 10.3390/nu15122776 (PMC10304786; doi:10.3390/nu15122776)
Supplement: Supplementary file 1 [file nutrients-15-02776-s001.zip › SUPPLE table.pdf]

Supplement Table S1. HPLC condition for analysis of ginsenoside Rg1, Rb1 and Rg3 in PG.

HPLC condition	Condition 1		
Column	C18 column (250 nm × 4.6 mm, 5 μm)		
Column temp.	35 °C		
Flow rate	1 mL/min		
Wave length	203 nm		
Injection volume	10 μL		
Mobile solvent	A: Acetonitrile<br>B: Water		
Mobile phase	Time (min)	A (%)	B (%)
	0	20	80
	40	60	40
	41	20	80
	45	20	80

Supplement Table S2. HPLC condition for analysis of tannic acid in DKL.

HPLC condition	Condition 2		
Column	Zorbax Eclipse Plus C18 column (250 nm × 4.6 mm, 5 μm)		
Column temp.	30 °C		
Flow rate	1 mL/min		
Wave length	280 nm		
Injection volume	10 μL		
Mobile solvent	A: Water<br>B: Acetonitrile		
Mobile phase	Time (min)	A (%)	B (%)
	0	95	5
	2	95	5

3	40	60
10	40	60
11	95	0
15	95	0

Supplement Table S3. Calibration data of ginsenoside Rg1, Rb1 and Rg3 in PG and tannic acid in DKL.

Compound	Linear range (mg/L)	Response Slope (a)	Response Factor (b)	Correlation coefficient (R^2)	LOD (mg/L)	LOQ (mg/L)
Ginsenoside Rg1	2.5 ~ 100	3283.2	-1139.6	0.9996	2.56	7.77
Ginsenoside Rb1	2.5 ~ 100	2323	-953.87	0.9996	2.43	7.36
Ginsenoside Rg3	2.5 ~ 100	3425.7	-464.03	0.9996	2.43	7.38
Tannic acid	18424.21	-736552.17	0.9987	49.68	150.55	

Supplement Table S4. Contents of ginsenoside Rg1, Rb1 and Rg3 in PG and tannic acid in DKL.

Sample	Contents of standards (%)		
	Ginsenoside Rg1	Ginsenoside Rb1	Ginsenoside Rg3
Panax ginseng (Ginseng Radix Red)	0.87	1.26	0.09
Panax ginseng: Diospyros kaki leaf (1:3)	0.25	0.23	0.03
Sample	Contents of standards (%)		

	Tannic acid
Diospyros kaki leaf	5.49
Panax ginseng: *Diospyros kaki* leaf (1:3)	5.06
